# p53: Pro-aging or pro-longevity?

**DOI:** 10.18632/aging.100178

**Published:** 2010-07-16

**Authors:** Peter L.J. de Keizer*, Rémi-Martin Laberge*, Judith Campisi

**Affiliations:** ^1^ Buck Institute for Age Research, Novato, CA 94945, USA; ^2^ Lawrence Berkley National Laboratory, Berkeley, CA 94720, USA; *These authors contributed equally to this work

p53 continues to surprise biologists.  For nearly a decade, it was thought to be an oncogene [1,2], only to be subsequently declared a potent tumor suppressor [3,4]. Initially characterized as a transcriptional activator, we now know p53 is also a transcriptional repressor [5].  And just as it seemed p53 activities were confined to the nucleus, it became apparent that p53 also functioned in the cytoplasm to regulate mitochondrial responses [6,7].  As a tumor suppressor and regulator of hundreds of genes [5], it was perhaps not surprising that p53 was shown to regulate numerous cellular processes related to cancer -- cell cycle progression, apoptosis, cellular senescence and DNA repair, among others.  It was another surprise, however, to learn that p53 might also regulate aging.

Half a dozen or so years ago, two landmark studies showed that, in mice, the constitutive expression of certain p53 mutants or naturally occurring isoforms resulted in chronically elevated p53 activity.  These transgenic mice were extraordinarily cancer resistant -- but they showed multiple signs of accelerated aging and died prematurely [8,9].  This pro-aging activity of p53 was thought to result from chronic p53-dependent apoptosis and/or senescence, resulting in cancer-resistance at the price of tissue atrophy or dysfunction [10,11]. Shortly thereafter, though, mice were engineered with extra copies of the wild-type p53 gene, and so they showed elevated p53 activity but in a normally regulated manner.  These mice were also extraordinarily resistant to cancer, but in this case they showed no signs of accelerated aging and had a normal life span [12].  Further, transgenic mice that overexpressed regulated p53 together with its upstream regulator ARF (p19) were not only cancer resistant but they lived significantly longer than wild-type controls [13].  In these models, the regulated hyperactive p53 activity was shown to reduce age-associated DNA damage and the accumulation of damaged cells.

Together, these studies indicate that p53 can promote or retard aging, depending on the context of its regulation and activity.

One obvious mechanism by which p53 might exert both its pro-aging and pro-longevity effects is by driving cell fate decisions.  As a pro-aging determinant and as discussed above, p53 might drive excessive apoptosis and/or cellular senescence.  These cell fates can, in turn, cause tissue atrophy and degeneration (apoptosis) and loss of tissue renewal or regenerative capacity (senescence).  As a pro-longevity determinant, p53 might eliminate damaged or dysfunctional cells (apoptosis) or prevent their proliferation and hence their ability to form tumors (senescence).

A perhaps less obvious mechanism by which p53 might promote or retard aging is by altering the systemic or local tissue milieu.  One potentially important p53 target in this regard is the insulin/insulin-like growth factor (IGF)-1 signaling (IIS) pathway.  IIS and one of its major intracellular targets, the mTOR pathway, drive aging in diverse species, ranging from yeast to mice [14].  In general, high IIS/mTOR activity is associated with cell proliferation, growth and aging, whereas low IIS/mTOR activity is associated with somatic maintenance and longevity.  In addition, p53 is regulated, directly and indirectly (through MDM2), by another major component of IIS signaling, the PKB/AKT kinase [15].  PKB/AKT signaling in turn is also both pro-aging (through the NF-kB transcription factor) and pro-longevity (through FOXO transcription factors) [16].

As is the case for all complex pathways, the precise phenotypes that are elicited by IIS/mTOR depend on the strengths of the activating or repressing signals, and on physiological context.  As a pro-aging determinant, p53 might stimulate IIS; conversely, as a pro-longevity determinant, it might reduce IIS.  So, what is the status of IIS in mice with elevated p53 activity?  Inconsistently, higher levels of circulating IGF-1 and tissue-associated IIS are present in both a short- [9] and long-lived [17] transgenic mouse with elevated p53 activity.  Moreover, a second short-lived hyper-p53 mouse model showed reduced IIS, at least in the mammary gland [18].  Further, IGFBP-3, a secreted IGF-1 binding protein that inhibits IGF-1 signaling, is a classic target of p53 transactivation activity [19].  Clearly, whether and to what extent the effects of p53 on aging and longevity are mediated by IIS must be determined in each of the mouse models, taking into account the multiple ways in which IIS activity can be modulated.

A second potentially important p53 target is the senescence-associated secretory phenotype (SASP).  As discussed above, p53 is an important regulator of cellular senescence [20], the essentially irreversible arrest of cell proliferation that occurs in response to potentially oncogenic stresses [21].  We recently showed that senescent cells secrete a plethora of biologically active molecules that can alter the systemic or local tissue milieu [22,23].  Of particular significance, p53 restrained the SASP [22].  That is, compared to wild-type cells, cells that lacked p53 function secreted markedly higher levels of most of the SASP components.

A striking feature of the SASP is the prevalence of pro-inflammatory cytokines [24,25].  Low level, chronic inflammation increases with age and is a cause or substantial contributor to virtually all of the major age-related diseases [26-29].  The source of this inflammation is not clear, but one possibility is that it derives at least in part from senescent cells, which increase with age [30].  It is tempting to speculate, then, that p53 might have pro-longevity effects not only because it suppresses tumorigenesis, but also because it keeps in check inflammation driven by senescent cells.

It is evident now that p53 can be either pro-aging or pro-longevity, depending on the physiological context.  The apparent paradox of how p53 modulates life span will undoubtedly resolve as we understand in greater detail how p53 and its activities impact specific aging phenotypes.  And in this regard, p53 will likely continue to surprise biologists.
